# Massive‐scale genomic analysis reveals SARS‐CoV‐2 mutation characteristics and evolutionary trends

**DOI:** 10.1002/mlf2.12040

**Published:** 2022-09-26

**Authors:** Yamin Sun, Min Wang, Wenchao Lin, Wei Dong, Jianguo Xu

**Affiliations:** ^1^ Research Institute of Public Health Nankai University Tianjin China; ^2^ TEDA Institute of Biological Sciences and Biotechnology Nankai University Tianjin China; ^3^ Engineering and Research Center for Microbial Functional Genomics and Detection, Ministry of Education Nankai University Tianjin China; ^4^ State Key Laboratory for Infectious Disease Prevention and Control, Chinese Center for Disease Control and Prevention National Institute for Communicable Disease Control and Prevention Beijing China; ^5^ Research Units of Discovery of Unknown Bacteria and Function Chinese Academy of Medical Sciences Beijing China; ^6^ Danaher Diagnostic Platform USA

**Keywords:** de novo mutation, evolutionary trends, mutation characteristics, mutation frequency, SARS‐CoV‐2

## Abstract

The severe acute respiratory syndrome coronavirus 2 (SARS‐CoV‐2) pandemic resulted in significant societal costs. Hence, an in‐depth understanding of SARS‐CoV‐2 virus mutation and its evolution will help determine the direction of the COVID‐19 pandemic. In this study, we identified 296,728 de novo mutations in more than 2,800,000 high‐quality SARS‐CoV‐2 genomes. All possible factors affecting the mutation frequency of SARS‐CoV‐2 in human hosts were analyzed, including zinc finger antiviral proteins, sequence context, amino acid change, and translation efficiency. As a result, we proposed that when adenine (A) and tyrosine (T) bases are in the context of AM (M stands for adenine or cytosine) or TA motif, A or T base has lower mutation frequency. Furthermore, we hypothesized that translation efficiency can affect the mutation frequency of the third position of the codon by the selection, which explains why SARS‐CoV‐2 prefers AT3 codons usage. In addition, we found a host‐specific asymmetric dinucleotide mutation frequency in the SARS‐CoV‐2 genome, which provides a new basis for determining the origin of the SARS‐CoV‐2. Finally, we summarize all possible factors affecting mutation frequency and provide insights into the mutation characteristics and evolutionary trends of SARS‐CoV‐2.

## Impact statement

In this study, we analyzed the possible factors affecting mutations in more than 2,800,000 high‐quality severe acute respiratory syndrome coronavirus 2 (SARS‐CoV‐2) genomes. To our knowledge we are the first to propose that when the A or T base conforms to AM or TA motif, the A or T base has a lower mutation frequency; and subsequently, translation efficiency can affect the mutation frequency from C/G to A/T on the third position of the codon by the selection. We found significant host‐specific asymmetric mutations at dinucleotide sites. In addition, we also identified the characteristics of SARS‐CoV‐2 mutations and hypothesized the evolutionary trends of the virus in the human host. These findings are valuable for predicting the development of the COVID‐19 pandemic and bring to light new hypotheses regarding the origin of SARS‐CoV‐2.

## INTRODUCTION

Severe acute respiratory syndrome coronavirus 2 (SARS‐CoV‐2), the causative agent of the ongoing SARS‐CoV‐2 pandemic, is a virus that belongs to the *Sarbecovirus* genus of the *Coronaviridae* family^1^. The SARS‐CoV‐2 outbreak rapidly spread worldwide^2^, causing an estimated 526,558,033 confirmed cases and 6,287,117 deaths, as reported by World Health Organization by May 23, 2022^3^. During the past 2 years, since the start of the COVID‐19 pandemic, a high number of new variants emerged from the more than 6 million genomes reported by January 18, 2022. The number of SARS‐COV‐2 viral genome sequences obtained exceeds the total number of other virus genomes and provides an extensive record of SARS‐CoV‐2 evolution during the pandemic. Accordingly, the analysis of SARS‐CoV‐2 genome sequences is of great significance to understand viral mutation and evolutionary trends.

Genomic mutations occurring in a viral sequence can be classified as beneficial, neutral, or deleterious mutations. Beneficial mutations are those increasing the fitness of the virus that usually help spread and replication relative to other strains. On the contrary, deleterious mutations hamper efficient replication and transmission, and will thus likely tend to disappear from the viral gene pool^4^, ^5^. During SARS‐CoV‐2 evolution, several significant mutations were identified^6^, ^7^, ^8^, ^9^. These mutations are important for the evolution of SARS‐CoV‐2 and the formation of new variants, including the D614G and N501Y mutations^10^, ^11^, ^12^. Hence, analyzing the occurrence, accumulation, and proportion of each mutation is of great value to determine the impact of these mutations on viral evolution^4^, ^13^, ^14^.

In genetics, de novo mutations (DNMs) refer to genetic variants that develop for the first time within a viral family^15^, ^16^. In humans, germline DNMs not only drive the evolution of our species but also represent an important cause of genetic disease^17^. To understand the evolutionary trends of viruses, it is extremely important to detect and analyze DNMs. Previous studies used within‐host variation detection to identify DNMs in SARS‐CoV‐2^18^, ^19^. While this method can accurately detect DNMs, it provides limited information about their characteristics and does not contribute to a deep comprehensive understanding of SARS‐CoV‐2 evolutionary trends.

An organism's mutation spectrum reflects the rate of different mutation types in different genome sites. The mutation spectrum of SARS‐CoV‐2 has been previously investigated across several studies, which showed a predominance of C to U substitutions, with additional high rates of G to U^20^, ^21^, ^22^, ^23^. However, the reasons behind these observations remain unknown. Current hypotheses suggest that high G to U substitution rates are associated with reactive oxygen species (ROS) that causes guanine oxidation to 8‐oxo‐7,8‐dihydroguanine (8‐oxoguanine). In turn, the 8‐oxoguanine can pair with adenine, ultimately causing a G to U transversion^24^. Previous studies have shown that two host RNA‐editing families can affect the SARS‐CoV‐2 mutational spectrum, specifically the cytosine deaminase enzymes (APOBECs), which cause C to U transitions^25^, ^26^, and adenosine deaminase acting on RNA (ADAR) enzymes that lead to adenosine‐to‐inosine mutations (A>G/U>C mutations)^27^. In these processes, zinc finger antiviral proteins (ZAPs) bind CpG dinucleotides in single‐stranded RNA and then recruit the RNA processing exosome for targeted degradation. In this way, ZAP is able to deplete CpG dinucleotides in viral genomes that infect mammalian species, such as SARS‐CoV‐2^28^, ^29^. The mutation spectrum is often used to study the origin of SARS‐CoV‐2, but there are different perspectives on whether this spectrum is host‐ or virus‐specific. Some speculated that the mutation spectrum of the viral genome is reflective of its evolution in different hosts, and can therefore be used to infer cross‐species transmission^30^. Alternatively, substitution patterns may be more virus‐specific than host‐specific, questioning the impact of cellular antiviral mechanisms on the substitution spectra of coronaviruses^28^, ^31^.

To address these questions, we identified 296,728 high‐quality DNMs in this study and analyzed all possible factors affecting their distribution and characteristics. In addition to viral intrinsic mutational biases, the ZAP enzymes, sequence context, amino acid changes, and translation efficiency can affect the mutation frequency of SARS‐CoV‐2. Here, we show the effect of the AM and TA motifs and translation efficiency on DNMs for the first time. In addition, we found a host‐specific asymmetric dinucleotides mutation rate in the SARS‐CoV‐2 genome. Our results show that, in the ancestral host, the dinucleotides mutation asymmetry was affected by the ZAP and APOBEC enzymes. In contrast, in the human host, the dinucleotides mutation asymmetry was affected by ZAP, AM, and TA motifs. Accordingly, our study provides new light on the origin of SARS‐CoV‐2.

## RESULTS

### A descriptive analysis of the spatio‐temporal distribution of all DNMs

In this study, we identified a total of 296,728 high‐quality DNMs in more than 2,800,000 high‐quality SARS‐CoV‐2 genomes. To understand the mutation characteristics of SARS‐CoV‐2, we analyzed the distribution of SARS‐CoV‐2's DNMs in the genome, including their spatio‐temporal distribution.

**Figure 1:**
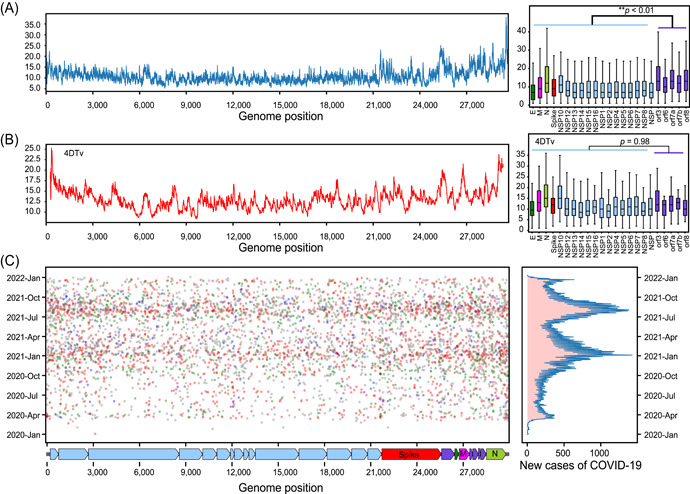
Distribution of de novo mutations (DNMs). (A) The distribution of DNMs in the whole genome. The *X*‐axis represents the positions of the genome while the *Y*‐axis shows the number of detected DNMs (window size 100 bp). The average number of detected DNMs in different genes is shown on the right side. (B) The distribution of DNMs on the 4DTv. The *X*‐axis represents the genome position while the *Y*‐axis shows the average number of DNMs detected at 4DTv sites (widow size 100 bp). On the right side is shown the average number of DNMs detected at each 4DTv site on different genes. (C) The temporal distribution of DNMs, with the genome location on the *X*‐axis and the time of detection on the *Y*‐axis. Each point represents a DNM. Different colors represent different countries, red points represent the United States and green points represent the United Kingdom. The graph on the right‐hand side shows the distribution curve of new cases of COVID‐19 world wide. E, gene encoding envelope protein; M, gene encoding membrane protein; N, gene encoding nucleocapsid protein; NSP, non‐structure protein.

We calculated the mutation frequency (average number of DNMs detected per base pair) of different genomic regions. The results showed that the genes encoding accessory proteins (orf3, orf6, orf7a, orf7b, and orf8) have a higher mutation frequency than those encoding structural proteins (e.g., spike protein, envelope protein, membrane protein, and nucleocapsid protein) and the *orf1ab* gene (encoding nonstructural proteins 1–16) (*p* < 0.01, *t*‐test) (Figure 1A). We further analyzed the frequency of Fourfold Degenerate Synonymous Site (4DTv) mutations across the whole genome. Our results showed that the mutation frequency at 4DTv sites is similar in different genes (Figure 1B). The accessory proteins have a more similar mutation frequency at all sites compared to 4DTv sites (Figure S1), and structural proteins and ORF1ab have lower mutation frequencies at all sites compared to 4DTv sites. These results imply purifying selection on structural proteins and ORF1ab. A lower mutation frequency was detected on the Spike protein, which accommodated a large number of mutations found in major SARS‐CoV‐2 variants, including Alpha and Omicron^32^, ^33^. These results suggest that mutation accumulation does not correlate with their respective frequency of occurrence in short‐term evolution.

We next evaluated the spatial distribution of DNMs and identified 103,281 DNMs in SARS‐CoV‐2 viral genomes isolated from the United States, which accounted for 34.81% of the total DNMs found in our study. In addition, we uncovered a total of 33,397 DNMs in genomes isolated from the United Kingdom, accounting for 11.26%. A total of 800,000 genomes from the United States and 820,000 genomes from the United Kingdom were used to identify DNMs. Next, we analyzed the average number of DNMs per genome across different countries and found that an average of 0.05 and 0.12 DNMs per genome were detected in the United Kingdom and the United States, respectively, which was highly similar to the average number (0.11) of DNMs detected in the world. This indicates that although the total number of DNMs detected in the United Kingdom and the United States is very high, the average number of DNMs detected per genome is normal. Thus, we speculate that the larger total number of DNMs detected in the United States and the United Kingdom may reflect the large genome sequencing efforts of these two countries. In addition, the larger number of DNMs detected in the United States and the United Kingdom may result from high traveling activities to these countries from various other countries and regions of the world, which could bring in a variety of DNMs. The temporal distribution of DNMs showed that the largest number was detected in January 2021 and August 2021, which was consistent with the spread of the alpha and delta variants, respectively (Figure 1C). This indicates that higher transmission frequencies within the human population lead to a higher number of DNMs, which increase the chances of the emergence of new variants.

### Sequence context affects the mutation frequency of SARS‐CoV‐2

**Figure 2:**
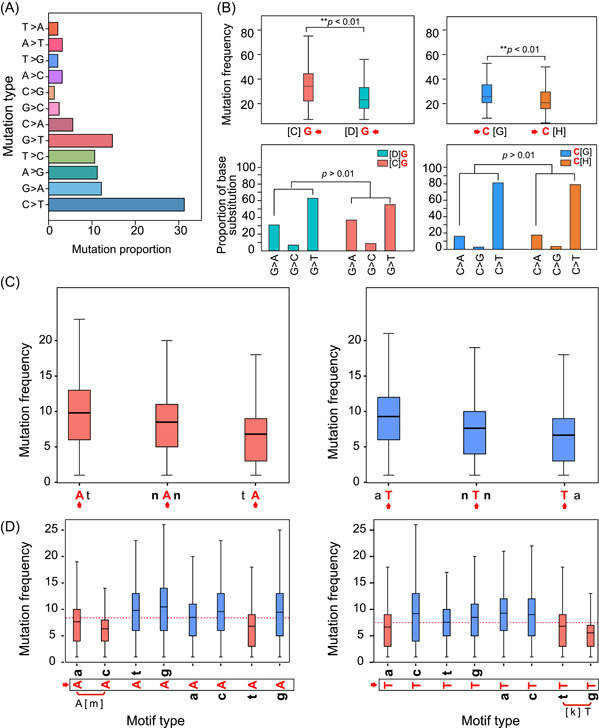
Mutation spectra and context sequences. (A) The whole‐genome mutation spectrum of SARS‐CoV‐2, that is, the proportion of 12 base substitution types. The *X*‐axis represents the proportion of 12 base substitution types, while the *Y*‐axis shows the 12 base substitution types. (B) The upper part is the frequency of G mutations (DG, D = A, T, G) and C mutations (CH, H = A, T, C) in CG and non‐CG motifs. *Y*‐axis represents the mutation frequencies. The lower part is the proportion of base substitution types of G mutations (DG, D = A, T, G) and C mutations (CH, H = A, T, C) in CG and non‐CG motifs. (C) The mutation frequencies of A and T bases on AT motif, TA motif, and the whole genome. The *X*‐axis represents the locus of A or T bases. nAn and nTn mean all the A or T bases of the whole genome, uppercase letters represent the bases used to calculate the mutation frequency and lowercase letters represent the context sequence. *Y*‐axis represents the mutation frequency. The A and T bases have higher average mutation frequencies when they conform to AT motif, and the lowest average mutation frequencies when they conform to the TA motif. (D) The mutation frequencies of A and T bases across 16 sequence motifs. The nucleotide bases marked with red arrows are used to calculate mutation frequency, and lowercase letters represent the context sequence. The average mutation frequency of A or T bases at the whole genome level is shown in a dotted line. SARS‐CoV‐2, severe acute respiratory syndrome coronavirus 2.

We calculated the frequencies of 12 base substitution types in 296,728 DNMs, and normalized the count by the number of genome‐wide A, T, C, and G. This allowed us to obtain the whole‐genome mutation spectrum of SARS‐CoV‐2, which showed a high frequency of C>T and G>T (Figure 2A), as previously reported^20^, ^23^, ^28^.

To determine the impact of sequence context on the mutation of SARS‐CoV‐2, we counted the mutation frequency of 12 different dinucleotides (CG, GC, AT, TA, TC, CT, AG, GA, CA, AC, TG, and GT). If meaningless, then each of the six dinucleotide pairs should have the same mutation frequency (CG=GC, CT=TC, CA=AC, GA=AG, GT=TG, AT=TA). However, our results showed CG dinucleotides have a significantly higher mutation rate than GC dinucleotides (*p* < 0.01, *t*‐test) (Figure S2 and Table 1), which is consistent with previous reports^34^, ^35^, ^36^. Since ZAP is able to specifically bind CpG dinucleotides in single‐stranded RNA for targeted degradation, a reduction in the number of CG dinucleotides likely reflects an evasion mechanism resulting from virus evolution. Contrastingly, base substitutions between CG and non‐CG motifs showed no differences, suggesting that ZAP only affected the mutation frequency, not the base substitution types (Figure 2B). This result is consistent with selection against CpG, mechanistically mediated by ZAP.

**Table 1:** Mutation frequencies in 12 dinucleotide types in the different hosts.

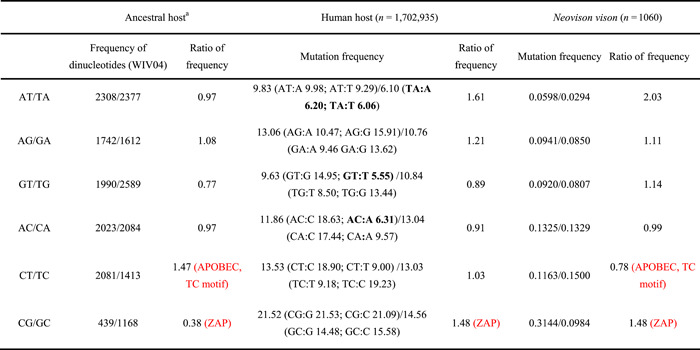

^a^ Sequence characteristics of the first severe acute respiratory syndrome coronavirus 2 (SARS‐CoV‐2) genome isolated from the human host represent the mutation characteristics of the ancestral host. The enzymes that affect the dinucleotide asymmetry of SARS‐COV‐2 are indicated in red in brackets. The motifs with base mutation frequency lower than the genome average mutation frequency are shown in bold.

Surprisingly, we also found asymmetric mutation frequency in other dinucleotide pairs, especially AT and TA (*p* < 0.01, *t*‐test) (Figure S2 and Table 1). AT dinucleotides have a higher mutation frequency than TA dinucleotides, which has not been previously reported. To explore the driving mechanism of this asymmetry, we compared the mutation frequencies of A and T bases on AT motif, TA motif, and the whole genome, respectively (Figure 2C). We found that both A and T bases had the highest mutation frequency on AT motif and the lowest mutation frequency on the TA motif. The above results imply that the higher mutation frequencies of A and T bases on AT motif results in the higher mutation frequency of AT dinucleotides. By the same token, lower mutation frequencies of A and T bases on the TA motif lead to lower mutation frequency of TA dinucleotides. Since the mutation frequencies of A and T bases on AT and TA motifs are different, the mutation frequency of AT and TA dinucleotides are asymmetric. Moreover, this also shows that sequence context can affect the base mutation frequency.

We next investigated if there is another motif affecting the base mutation frequency, in addition to AT and TA motifs. So, we compared the mutation frequencies of A base on eight dinucleotide motif types (**A**a, **At**, **A**c, **A**g, a**A**, t**A**, c**A**, and g**A**, uppercase bold letters represent the bases used to calculate the mutation frequency and lowercase letters represent the context sequence) and T base on eight dinucleotide motif types (a**T**, t**T**, c**T**, g**T**, **T**a, **T**t, **T**c, and **T**g). Compared with the average mutation frequency of A and T bases on the whole genome (nAn and nTn, n = A, T, C, and G), we found that when the downstream context sequence of the **A** base is a or c base (**A**m motif, m = a or c), this **A** base has a low mutation frequency. Similarly, when the upstream context sequence of the **T** base is t or g bases (k**T** motif, k = t or g), this **T** base has a low mutation frequency (Figure 2D). Moreover, it should be noted that the motif sequences **A**m and k**T** are reversely complementary to each other, which indicates that the two motif sequences act on the positive‐ and negative‐sense strands of the RNA virus through a similar mechanism.

The above results show that when the sequence conforms to the t**A** and **A**m motif (reverse complement **Ta** and k**T**), the A (or T) base also has a lower mutation frequency. This raised the question of why the mutation of AT/TA dinucleotides is more asymmetrical than AC/CA and GT/TG. We speculate that when the sequence conforms to Am motif (reverse complement kT motif), only the mutation frequency of one nucleotide base is reduced: A base in the upstream of **A**a and **A**c dinucleotide motif, or T in the downstream of g**T** and t**T** dinucleotide motif. However, when the sequence conforms to the **TA** motif (**T**a or t**A**), the mutation frequency of both **A** and **T** bases is reduced, resulting in more asymmetry of AT/TA compared with other dinucleotide pairs.

To determine the effect of codon position on dinucleotide mutation asymmetries, we calculated the dinucleotide mutation frequencies of three codon phases: 1st + 2nd, 2nd + 3rd, and 3rd + 1st (Table S2). The results showed that AT/TA dinucleotides were more asymmetric in the 1st + 2nd and 3rd + 1st codon phase; CG/GC dinucleotides showed more asymmetry in the 2nd + 3rd and 3rd + 1st codon phase. These results show that the dinucleotide asymmetry is different in different codon phases.

### Amino acid changes affect the mutation frequency of SARS‐CoV‐2

**Figure 3:**
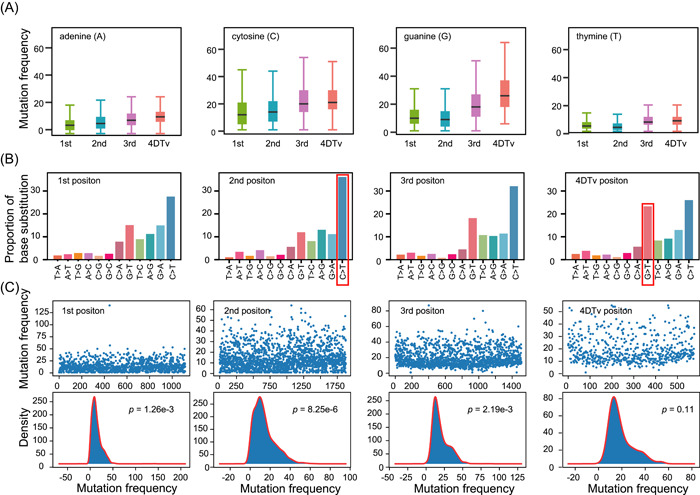
The mutation characteristics at the first, second, and third codon positions, as well as at 4DTv. (A) The mutation frequency of the first, second, and third codon, and 4DTv. (B) The mutation spectrum. The *Y*‐axis represents the proportion of 12 base substitution types, while the *X*‐axis shows the 12 base substitution types. (C) The distribution of C>T at the first, second, and third codon, and 4DTv. The upper part is the distribution of mutation frequency, *X*‐axis shows the mutation position (e.g., the number 200 represents the 200th C nucleotide base at the 1st of the reference genome), and the *Y*‐axis represents the mutation frequency. The lower part is histoplot of distribution of DNM mutation frequency. DNM, de novo mutation.

The coding region of the SARS‐CoV‐2 genome comprises more than 97.51% of the total genome. Of the 296,728 identified DNMs, 15,196 were found in the noncoding region (20.59 DNMs/bp on average) and 281,532 in the coding region (11.00 DNMs/bp). Hence, our findings demonstrate that the coding region has a lower mutation rate than the noncoding region. However, mutations in the coding region might cause amino acid changes, whereby we sought to determine the relationship between amino acid changes and mutation frequency. Accordingly, we calculated the mutation frequency, mutation spectrum, and distribution of mutations at the first, second, and third codon positions, as well as at 4DTv. We found that the mutation frequency at the third codon position and 4DTv sites was higher than that of the first and second codon positions (Figure 3A). This is likely due to purifying selection, as mutations at the first and second codon positions could result in amino acid changes that might affect viral replication and transmission.

From the mutation spectrum, we found a higher C>T mutation rate at the second codon position (Figure 3B). NTN codons (where N = any nucleotide) usually code for hydrophobic amino acids, while NCN amino acid residues are smaller in size and moderate in hydropathy. We uncovered a total of 179,227 nonsynonymous DNMs, of which 101,120 and 69,458 resulted in hydrophobic and hydrophilic amino acids, respectively. These results may suggest that SARS‐CoV‐2 is evolving toward the accumulation of more hydrophobic amino acids.

If nucleotide bases at a specific codon position have similar mutation frequencies, then the mutation number of each nucleotide base is random, and therefore the mutation frequency (mutation number at a single site) should follow a Poisson distribution^37^. In this study, we analyzed the distribution of C>T at the first, second, and third codon, and 4DTv, respectively, and used a Kolmogorov–Smirnov test (K–S test) to determine whether it follows a Poisson distribution. The results showed that compared with 4DTv, the mutation frequency at the first, second, and third codon positions does not follow a Poisson distribution, indicating that the mutation frequency is likely constrained by amino acid changes (Figure 3C). The above results indicate that the amino acid change can affect the mutation frequency of SARS‐CoV‐2, which may be caused by positive selection.

### Translation efficiency affects the mutation frequency of SARS‐CoV‐2

**Figure 4:**
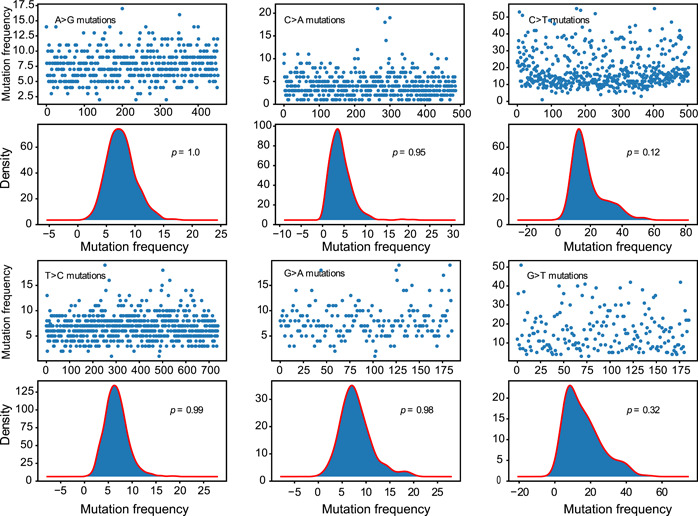
The mutation frequency distribution of non‐CG and non‐AM, and non‐TA sites at 4DTv. The upper part is the distribution of DNM mutation frequency, *X*‐axis shows the mutation position (e.g., the number 200 represents the 200th non‐CG, non‐AM, and non‐TA cytosine nucleotide base at 4DTv of the reference genome), and *Y*‐axis represents the mutation frequency. The lower part is histoplot of distribution of DNM mutation frequency. DNM, de novo mutation.

We next evaluated whether there were other unknown factors affecting the mutation frequency. Assuming no other factors influence the mutation rate, 4DTv site nucleotide bases with non‐CG, non‐AM, and non‐TA motifs should follow a Poisson distribution. We found that the 12 nucleotide base substitution types conformed to a Poisson distribution. However, C>T and G>T mutations showed a lower *p* value when compared to the *p* value of the K–S test above, suggesting that other factors affect C or G to T mutations at 4DTv (Figures 4 and S3).

**Figure 5:**
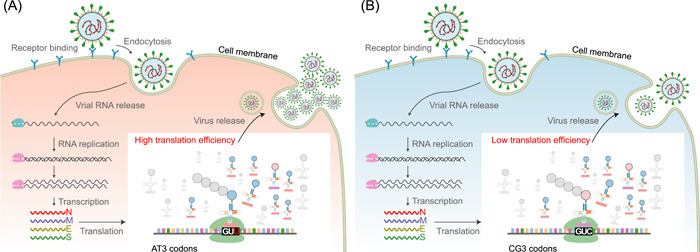
The life cycle of SARS‐CoV‐2 in host cells. When CG3 codons mutate into AT3, the virus gains higher translation efficiency and replication producing more viruses. (A) AT3 codons have high translation efficiency. (B) CG3 codons have low translation efficiency. SARS‐CoV‐2, severe acute respiratory syndrome coronavirus 2.

Therefore, we then calculated the frequency of SARS‐CoV‐2 codon usage and found that >73% of codes include A/T ending codons (AT3 codons), while the T ending codons accounted for more than 44% of the total number of codons. This value is significantly higher than the average number of AT (62%) and T (32%) nucleotides in the genome, respectively (Figure S4). This observation demonstrates that SARS‐CoV‐2 preferentially uses AT3 codons, in particular T ending codons. Previous studies suggested that the increased expression of transfer RNA carrying AT3 codons in SARS‐CoV‐2 patients could reduce the stability of the host mRNAs and affect the synthesis of host proteins^14^, ^38^. This implies that, while SARS‐CoV‐2 limits the host protein synthesis, its own translation efficiency of CG3 codons is also limited. When the third codon position is mutated from C/G to A/T, the translation efficiency of the virus increases, and the virus replicates more efficiently in the host. Within‐host virus replication is an exponential growth process, whereby a slight improvement in translation efficiency can greatly impact viral load. Therefore, translation efficiency may affect the mutation frequency of third codon position, which usually mutates into A/T, especially the T base (Figure 5).

The mutation frequency of 5′‐untranslated region (5′‐UTR) and 3′‐UTR are not affected by translation efficiency and amino acid changes. We next compared the mutation spectrum of 4DTv, 5′‐UTR, and 3′‐UTR of non‐CG, non‐AM, and non‐TA. Our results showed that the proportion of C and G mutations into T was highest at 4DTv sites (Table S1). This further demonstrated that translation efficiency impacts the mutation frequency of bases at the third codon position. The above results suggest that translation efficiency may affect the mutation frequency of C/G to A/T at the third codon position by the selection, consequently increasing AT3 codon usage.

### Dinucleotide asymmetric mutations of SARS‐CoV‐2 are host specific

If the mutation rate on the ancestral host is not affected by the sequence context, then it is expected that dinucleotide frequencies in the first SARS‐CoV‐2 genome isolated from the human host were randomly distributed; that is, the frequency of CG dinucleotides should be equal to that of GC dinucleotides, which is equal to the percentage of C content in the genome multiplied by the percentage of G content (CG% = GC% = C%*G%). The remaining five dinucleotide pairs should also conform to this expectation. To determine whether the SARS‐CoV‐2 mutation rate in the ancestral host was affected by sequence context, we calculated the ratio of six dinucleotide pairs in the SARS‐CoV‐2 reference genome (which represents the ancestral host). The results showed that the proportion of CG/GC was significantly asymmetrical (CG/GC = 0.38), suggesting that a ZAP enzyme existed in the ancestral host of SARS‐CoV‐2. Contrastingly, the proportion of AT/TA was 0.97, which suggests no mutation asymmetry exists in AT and TA dinucleotides in the ancestral SARS‐CoV‐2 host (Table 1). This result also indicates that the AT/TA mutation asymmetries of SARS‐COV‐2 are different between the ancestral and human hosts. To understand whether this asymmetry only exists in human hosts, we identified and analyzed DNMs from 1060 SARS‐CoV‐2 genome sequences from *Neovison vison* available in public databases and calculated the frequency of mutations in AT and TA dinucleotides. The results showed that the AT/TA ratio was also asymmetric in *N. vison*, whereby we can conclude that AT/TA mutation asymmetries do not exist only in the human host. In addition, we found that CT/TC and GT/TG ratios were 1.47 and 0.77 in the SARS‐CoV‐2 reference genome, respectively, pointing to asymmetries in CT/TC and GT/TG in the ancestral SARS‐CoV‐2 host. The CT/TC mutation asymmetry may be affected by the APOBEC enzyme (TC motif), while that of GT/TG may be affected by other unknown factors (Table 1). The aforementioned results show that dinucleotide mutation asymmetries have different profiles in different hosts and may help unveil the origin of SARS‐CoV‐2.

## DISCUSSION

In this study, we analyzed the factors that might affect the SARS‐CoV‐2 mutation rate^1^: The intrinsic mutational biases, which may be caused by RNA polymerase or other processes related to viral replication. This factor produces similar effects on all nucleotide bases across the genome, making it challenging to determine specific effects using bioinformatics. For example, the ROS enzyme causes guanine oxidation to 8‐oxo‐7,8‐dihydroguanine (8‐oxoguanine), which can pair with adenine, ultimately causing a higher rate of G to U transversions^24^, ^39^, ^40^. This factor impacts all G>U in the genome in a motif or position‐independent fashion. Hence, it is difficult to analyze using computational approaches. Therefore, we evaluated factors impacting beyond position and motif specificity as intrinsic mutational biases^2^. Consistent with previous literature^28^, ^29^, we observed that the ZAP enzyme affects CG dinucleotides by specifically binding CpG dinucleotides in single‐stranded RNA and recruiting the RNA processing exosome for targeted degradation^3^. Furthermore, we proposed, for the first time that when A and T bases are in the context of AM (M stands for adenine or cytosine) or TA motif, A or T base has lower mutation frequency^4^. Additionally, the mutation frequency of the base at the first or second codon is lower than that of the third codon, which is likely due to purifying selection. As the mutations at the first and second codons could result in amino acid changes, virus replication and transmission might be affected.^5^ Finally, the AT3 codons have higher translation efficiency than CG3 codons, further favoring C/G nucleotide mutation to A/T at the third codon position.

In genetics, DNMs refer to genetic variants that develop for the first time within a viral family, and, in this study, the detected DNMs are not strictly in this sense, and were likely the result of selection^41^, ^42^. However, these DNMs affected by selection are closer to the true SARS‐COV‐2 mutations that reflect the virus evolutionary process, and therefore these DNMs are more relevant for understanding SARS‐CoV‐2 evolution in the host. Among all the five factors here analyzed, intrinsic mutational biases act on the factors causing mutational bias themselves, while ZAP enzyme, codon position, and translation efficiency impact mutations after they arise through selection. The phenomenon of A or T having lower mutation frequencies in the context of AM and TA motifs cannot be strictly linked to a single factor, so we cannot determine whether it is driven by mutation or selection. However, these lower mutation frequencies are found in both positive and negative chains of SARS‐COV‐2, implying that it is connected to the viral replication process. Therefore, this factor tends to act on the virus mutation itself. Future studies will try to determine the possible factors causing this phenomenon.

In this study, we first reported the phenomenon of AT/TA dinucleotide mutation asymmetries but did not determine what caused the phenomenon. We initially assumed that ADARs act on RNA to convert adenosine into inosine in double‐stranded RNA regions. Since inosine is recognized as guanosine, the result of editing by ADARs is a A>G transition mutation. Previous studies have shown the impact of ADAR‐induced editing of minor viral RNA populations on the replication and transmission of SARS‐CoV‐2^27^. However, by comparing the mutation frequencies of A or T bases across 16 motif sequences, we found that A or T base in 6 specific sequences motif (t**A**, **A**c, **A**a, **T**a, t**T**, and g**T**) exhibited a lower mutation frequency than that in other 10 sequences motif (a**A**, c**A**, g**A**, **A**t, **A**g, a**T**, c**T**, **T**t, **T**c, and **T**g) (Figure 2D). According to Occam's razor principle, it is more likely for AM and TA motifs to reduce the mutation frequency of A or T through one or more factors. Therefore, this speculation means that the mutation asymmetries of AT/TA are caused by reducing the mutation frequency of TA dinucleotides, which is contrary to the speculation of ADARs enzyme.

Previous research shows that TA (UpA in RNA) dinucleotides have a higher mutation frequency in the genomes of most RNA virus groups^43^, ^44^, ^45^, while in the SARS‐COV‐2, TA dinucleotides have a lower mutation frequency, which is unusual. Therefore, in‐depth analysis of AT/TA dinucleotides mutation asymmetries in subsequent studies is of great value for understanding the evolution of SARS‐COV‐2.

Analysis of the mutation frequency of 4DTv site nucleotide bases with non‐CG, non‐AM, and non‐TA motifs revealed distributions of C>T and G>T mutation frequencies with heavier right tails, which seem to be a superimposition of two distributions. This phenomenon implies site‐specific selection; however, we could not identify a specific sequence motif, gene, or position that could drive these differences. Therefore, we hypothesize that an unknown factor drives this phenomenon.

The mutation spectrum is often used to study the origin of SARS‐CoV‐2, but there are different views on whether it is host‐ or virus‐specific. The intrinsic mutational biases are related to the virus's own genome and should thus be virus‐specific. In turn, the other four factors (ZAP, AM, and TA motifs, amino acid changes, and translation efficiency) are affected by the host and should thus be host‐specific. We found no differences between the mutation spectrum of humans and *N. vison* (*p* = 1, *t*‐test) (Figure S5), suggesting that on the short‐term evolutionary scale, the mutation spectrum of the virus is dominated by virus‐specific intrinsic mutational biases. Therefore, the use of the mutation spectrum is unreliable in short‐term evolutionary scales that allow studying the origin of the virus. In the long‐term, large‐scale evolutionary time, the influence of host selection pressures on mutation patterns will increase, and the mutation spectrum will then exhibit host‐specific signatures. Compared with the mutant spectrum, the dinucleotide mutation asymmetries found in this study seem to be host‐specific, which may help trace the origin of SARS‐CoV‐2.

In this study, DNM detection depends on the accuracy of the evolutionary tree. If the construction of the evolutionary tree is inaccurate, different genomes with the same DNMs will be separated into multiple evolutionary branches, and therefore defined as two or more DNMs. To avoid this, we analyzed the SARS‐CoV‐2 sequencing data separately by country, time, and evolutionary branch. This allows for revealing the probability of recurrent DNMs, which can be used to distinguish single DNMs. Some studies have characterized recurrent mutation in SARS‐COV‐2^46^. If recurrent mutations occur, the assignment of mutations present in >50% of a clade to the ancestor could lead to the missing of some DNMs. However, the probability of detecting a recurrent mutation in a small clade is very low. Among the 296,728 detected clades of DNMs, 99.43% clades were small (genomes no. <1000). For a ∼29 kb SARS‐COV‐2 genome, according to the mutation rate of 3 × 10^−3^, the probability of detecting the same position and the same mutation type in 1000 genomes within 2 years of evolution is also very low. Of course, we cannot ignore the impact of convergent evolution and selection on a few loci, which increases the probability of recurrence mutation at a few loci. However, in general, we believe that recurrent mutation has less impact on the accuracy of our current method. In future studies, we will try to reconstruct the subset of the evolutionary tree and merge the analysis information of mutation in the population to increase the accuracy of DNM detection.

Through DNM analysis, we characterized the mutation and evolutionary trend of SARS‐COV‐2. However, most of the detected DNMs in this study are near tips, and mutation accumulation is the key factor determining the direction of virus evolution. Future research should analyze the relationship between mutation characteristics and mutation accumulation of SARS‐COV‐2 to improve the understanding of the evolutionary trend of SARS‐COV‐2. In addition, compared with other early variants, Omicron has lighter clinical symptoms and different tropism; therefore, it will be important to compare the mutation profile of this variant with that of other variants.

## MATERIALS AND METHODS

### Data collection

SARS‐CoV‐2 sequences were retrieved from the Global Initiative on Sharing Avian *Influenza* Data initiative database (as of January 18, 2022, https://www.gisaid.org)^47^. Complete genomes with an N‐content lower than 0.01% and high coverage were selected for subsequent analysis. A multiple alignment using fast Fourier transform‐generated alignment of high‐coverage complete genome sequences was downloaded from the website.

### Mutation analysis

The complete SARS‐CoV‐2 genome isolate Wuhan‐Hu‐1 (NC_045512.2) was used as the reference genome; mutations in all other samples were compared to this reference isolate. Detected mutations were confirmed using Integrative Genomics Viewer^48^ and annotated with the SnpEff program^49^.

### Construction of phylogenetic tree

The amount of computation needed to construct an evolutionary tree for the 2.8 million genomes is substantial. Hence, to improve computational efficiency, SARS‐CoV‐2 genomes were classified by pangolin lineages using the pangoLEARN algorithm^50^. The 2.8 million genomes were divided into 1514 subsets according to their pangolin lineage. The RAxML^51^ software was used to determine the topological relationship between each subset according to their common mutations, and to construct the evolutionary tree as “root‐tree.” The maximum likelihood phylogenetic tree was constructed based on the general time reversible + invariant + gamma sites (GTR + I + G) model of nucleotide substitution with 1000 bootstrap replicates. After this, 1514 evolutionary trees were constructed as “branch‐trees” for the 1514 subsets trees using the FastTree^52^ software with Jukes‐Cantor model. Finally, “root‐tree” and “branch‐trees” were merged to generate the “final‐tree” by in‐house script. The flowchart of evolutionary tree construction is provided in Figure S6.

### DNM detection

The information on the distribution of each mutation in the different clades of the “final‐tree” was determined using an in‐house developed script. For each mutation, we step‐by‐step scan the “final‐tree” from root to tips to determine the proportion of mutation in each clade. When >50% of the genomes in a clade contained a particular mutation, we assumed the ancestor node of the clade containing the DNMs. To avoid the identification of inherited mutations as DNMs due to inaccurate terminal branching, we merged the DNMs that satisfy all the following conditions^1^: The same mutation type, such as C10029T (the 10,029th base position in the genome is mutated from C to T)^2^, the DNMs appear in the same clade and the clade size is <2000 genomes^3^; DNMs were isolated from the same country^4^; and time span of the DNMs is <6 months. We used these criteria given the very low probability of detecting multiple DNMs in the same country in 2000 genomes within 6 months. If the mutation rate of COVID‐19 is calculated as 3 × 10^−3^ nucleotide substitution per site per year, the probability of detecting the same DNM in 2000 genomes within 6 months should be: *p* = 0.009 (3 × 10^−3^ × 3 × 10^−3^ × 2000 × 0.5). To avoid the impact of sequencing errors on DNM detection, we filtered out DNMs based on a single genome. The flowchart of DNMs detection is provided in Figure S7.

## AUTHOR CONTRIBUTIONS

Yamin Sun was involved in data analysis, visualization, and writing original draft of the manuscript. Min Wang was involved in writing original draft. Wenchao Lin was involved in data analysis and visualization. Wei Dong was involved in software and data curation. Jianguo Xu was involved in supervision of this study, reviewing and editing the manuscript.

## ETHICS STATEMENT

This study has no animal or human experiments. There are no ethical issues involved.

## CONFLICT OF INTERESTS

The authors declare no conflict of interests.

## Supporting information

Supporting information.

## Data Availability

All scripts used to analyze the data and to generate the figures are available from figureShare (https://doi.org/10.6084/m9.figshare.19471571.v4). All data used to support the findings of this study are available in the aforementioned public databases.

## References

[1] Pal M , Berhanu G , Desalegn C , Kandi V . Severe acute respiratory syndrome coronavirus‐2 (SARS‐CoV‐2): an update. Cureus. 2020;12:e7423. 32337143 10.7759/cureus.7423 PMC7182166

[2] Zhu N , Zhang D , Wang W , Li X , Yang B , Song J , et al. A novel coronavirus from patients with pneumonia in China, 2019. N Engl J Med. 2020;382:727–33. 31978945 10.1056/NEJMoa2001017 PMC7092803

[3] World Health Organization (WHO) . *Weekly* epidemiological update on COVID. Available from: https://www.who.int/publications/m/item/weekly-epidemiological-update-on-covid-19-1-february-2022weekly-epidemiological-update-on-covid-19---24-august-2022

[4] Loewe L , Hill WG . The population genetics of mutations: good, bad and indifferent. Philos Trans R Soc Lond B Biol Sci. 2010;365:1153–67. 20308090 10.1098/rstb.2009.0317 PMC2871823

[5] Orr HA . The population genetics of beneficial mutations. Philos Trans R Soc Lond B Biol Sci. 2010;365:1195–201. 20308094 10.1098/rstb.2009.0282 PMC2871816

[6] Cheng XW , Li J , Zhang L , Hu WJ , Zong L , Xu X , et al. Identification of SARS‐CoV‐2 variants and their clinical significance in Hefei, China. Front Med. 2021;8:784632. 10.3389/fmed.2021.784632 PMC8784789 35083244

[7] Chen J , Wang R , Wang M , Wei GW . Mutations strengthened SARS‐CoV‐2 infectivity. J Mol Biol. 2020;432:5212–26. 32710986 10.1016/j.jmb.2020.07.009 PMC7375973

[8] Tian F , Tong B , Sun L , Shi S , Zheng B , Wang Z , et al. N501Y mutation of spike protein in SARS‐CoV‐2 strengthens its binding to receptor ACE2. eLife. 2021;10:e69091. 34414884 10.7554/eLife.69091 PMC8455130

[9] World Health Organization (WHO) . Tracking SARS‐CoV‐2 variants. Available from: https://www.who.int/en/activities/tracking-SARS-CoV-2-variants/

[10] Zhang L , Jackson CB , Mou H , Ojha A , Peng H , Quinlan BD , et al. SARS‐CoV‐2 spike‐protein D614G mutation increases virion spike density and infectivity. Nat Commun. 2020;11:6013. 33243994 10.1038/s41467-020-19808-4 PMC7693302

[11] Ali F , Kasry A , Amin M . The new SARS‐CoV‐2 strain shows a stronger binding affinity to ACE2 due to N501Y mutant. Med Drug Discovery. 2021;10:100086. 10.1016/j.medidd.2021.100086 PMC7923861 33681755

[12] Rahimi F , Abadi ATB . Implications of the emergence of a new variant of SARS‐CoV‐2, VUI‐202012/01. Arch Med Res. 2021;52:569–71. 33526352 10.1016/j.arcmed.2021.01.001 PMC7826083

[13] Valesano AL , Rumfelt KE , Dimcheff DE , Blair CN , Fitzsimmons WJ , Petrie JG , et al. Temporal dynamics of SARS‐CoV‐2 mutation accumulation within and across infected hosts. PLoS Pathog. 2021;17:e1009499. 33826681 10.1371/journal.ppat.1009499 PMC8055005

[14] Wang Y , Li J , Zhang L , Sun HX , Zhang Z , Xu J , et al. Plasma cell‐free RNA characteristics in COVID‐19 patients. Genome Res. 2022;32:228–41. 35064006 10.1101/gr.276175.121 PMC8805721

[15] Acuna‐Hidalgo R , Veltman JA , Hoischen A . New insights into the generation and role of de novo mutations in health and disease. Genome Biol. 2016;17:241. 27894357 10.1186/s13059-016-1110-1 PMC5125044

[16] Rambaut A , Loman N , Pybus O , Barclay W , Barrett J , Carabelli A , et al. Preliminary genomic characterisation of an emergent SARS‐CoV‐2 lineage in the UK defined by a novel set of spike mutations. 2020. Available from: https://virological.org/t/preliminary-genimic-characterrisation-of-on-emergent-sars-cov-2-lineage-in-the-uk-defined-by-a-novel-set-of-spike-mutations/563

[17] Rahbari R , Wuster A , Lindsay SJ , Hardwick RJ , Alexandrov LB , Turki SA , et al. Timing, rates and spectra of human germline mutation. Nat Genet. 2016;48:126–33. 26656846 10.1038/ng.3469 PMC4731925

[18] Al Khatib HA , Benslimane FM , Elbashir IE , Coyle PV , Al Maslamani MA , Al‐Khal A , et al. Within‐host diversity of SARS‐CoV‐2 in COVID‐19 patients with variable disease severities. Front Cell Infect Microbiol. 2020;10:575613. 33123498 10.3389/fcimb.2020.575613 PMC7572854

[19] Tonkin‐Hill G , Martincorena I , Amato R , Lawson AR , Gerstung M , Johnston I , et al. Patterns of within‐host genetic diversity in SARS‐CoV‐2. eLife. 2021;10:e66857. 34387545 10.7554/eLife.66857 PMC8363274

[20] Yi K , Kim SY , Bleazard T , Kim T , Youk J , Ju YS . Mutational spectrum of SARS‐CoV‐2 during the global pandemic. Exp Mol Med. 2021;53:1229–37. 34453107 10.1038/s12276-021-00658-z PMC8393781

[21] Rahman MS , Islam MR , Hoque MN , Alam A , Akther M , Puspo JA , et al. Comprehensive annotations of the mutational spectra of SARS‐CoV‐2 spike protein: a fast and accurate pipeline. Transbound Emerg Dis. 2021;68:1625–38. 32954666 10.1111/tbed.13834 PMC7646266

[22] Deng S , Xing K , He X . Mutation signatures inform the natural host of SARS‐CoV‐2. Natl Sci Rev. 2022;9:nwab220. 35211321 10.1093/nsr/nwab220 PMC8690307

[23] Elaswad A , Fawzy M , Basiouni S , Shehata AA . Mutational spectra of SARS‐CoV‐2 isolated from animals. PeerJ. 2020;8:e10609. 33384909 10.7717/peerj.10609 PMC7751428

[24] Suzuki T , Kamiya H . Mutations induced by 8‐hydroxyguanine (8‐oxo‐7,8‐dihydroguanine), a representative oxidized base, in mammalian cells. Genes Environ. 2017;39:2. 27980700 10.1186/s41021-016-0051-y PMC5131436

[25] Mourier T , Sadykov M , Carr MJ , Gonzalez G , Hall WW , Pain A . Host‐directed editing of the SARS‐CoV‐2 genome. Biochem Biophys Res Commun. 2021;538:35–9. 33234239 10.1016/j.bbrc.2020.10.092 PMC7643664

[26] Di Giorgio S , Martignano F , Torcia MG , Mattiuz G , Conticello SG . Evidence for host‐dependent RNA editing in the transcriptome of SARS‐CoV‐2. Sci Adv. 2020;6:eabb5813. 32596474 10.1126/sciadv.abb5813 PMC7299625

[27] Ringlander J , Fingal J , Kann H , Prakash K , Rydell G , Andersson M , et al. Impact of ADAR‐induced editing of minor viral RNA populations on replication and transmission of SARS‐CoV‐2. Proc Natl Acad Sci USA. 2022;119:e2112663119. 35064076 10.1073/pnas.2112663119 PMC8833170

[28] Forni D , Cagliani R , Pontremoli C , Clerici M , Sironi M . The substitution spectra of coronavirus genomes. Brief Bioinform. 2022;23:bbab382. 34518866 10.1093/bib/bbab382 PMC8499949

[29] Tonkin‐Hill G , Martincorena I , Amato R , Lawson A , Gerstung M , Johnston I , et al. Patterns of within‐host genetic diversity in SARS‐CoV‐2. elife. 2021;10:e66857. 34387545 10.7554/eLife.66857 PMC8363274

[30] Shan KJ , Wei C , Wang Y , Huan Q , Qian W . Host‐specific asymmetric accumulation of mutation types reveals that the origin of SARS‐CoV‐2 is consistent with a natural process. Innovation. 2021;2:100159. 34485968 10.1016/j.xinn.2021.100159 PMC8405235

[31] Klimczak LJ , Randall TA , Saini N , Li J‐L , Gordenin DA . Similarity between mutation spectra in hypermutated genomes of rubella virus and in SARS‐CoV‐2 genomes accumulated during the COVID‐19 pandemic. PLoS One. 2020;15:e0237689. 33006981 10.1371/journal.pone.0237689 PMC7531822

[32] Cheng XW , Li J , Zhang L , Hu W‐J , Zong L , Xu X , et al. Identification of SARS‐CoV‐2 variants and their clinical significance in Hefei, China. Front Med. 2021;8:784632. 10.3389/fmed.2021.784632 PMC8784789 35083244

[33] He X , Hong W , Pan X , Lu G , Wei X . SARS‐CoV‐2 Omicron variant: characteristics and prevention. MedComm. 2021;2:838–45. 34957469 10.1002/mco2.110 PMC8693031

[34] Zimmer MM , Kibe A , Rand U , Pekarek L , Ye L , Buck S , et al. The short isoform of the host antiviral protein ZAP acts as an inhibitor of SARS‐CoV‐2 programmed ribosomal frameshifting. Nat Commun. 2021;12:1–15. 34893599 10.1038/s41467-021-27431-0 PMC8664833

[35] Kumar A , Goyal N , Saranathan N , Dhamija S , Saraswat S , Menon MB , et al. The slowing rate of CpG depletion in SARS‐CoV‐2 genomes is consistent with adaptations to the human host. Mol Biol Evol. 2022;39:msac029. 35134218 10.1093/molbev/msac029 PMC8892944

[36] Wang Y , Mao J‐M , Wang G‐D , Luo Z‐P , Yang L , Yao Q , et al. Human SARS‐CoV‐2 has evolved to reduce CG dinucleotide in its open reading frames. Sci Rep. 2020;10:1–10. 32704018 10.1038/s41598-020-69342-y PMC7378049

[37] Hoffman JIE . The Poisson distribution. In: Teixeira RE , editor. Biostatistics for medical and biomedical practitioners. The Netherlands: Academic Press; 2015. p. 259–78.

[38] Hia F , Yang SF , Shichino Y , Yoshinaga M , Murakawa Y , Vandenbon A , et al. Codon bias confers stability to human mRNAs. EMBO Rep. 2019;20:e48220. 31482640 10.15252/embr.201948220 PMC6831995

[39] Poetsch AR . The genomics of oxidative DNA damage, repair, and resulting mutagenesis. Comput Struct Biotechnol J. 2020;18:207–19. 31993111 10.1016/j.csbj.2019.12.013 PMC6974700

[40] Giorgio M , Dellino GI , Gambino V , Roda N , Pelicci PG . On the epigenetic role of guanosine oxidation. Redox Biol. 2020;29:101398. 31926624 10.1016/j.redox.2019.101398 PMC6926346

[41] Kustin T , Stern A . Biased mutation and selection in RNA viruses. Mol Biol Evol. 2021;38:575–88. 32986832 10.1093/molbev/msaa247 PMC7543401

[42] Sane M , Diwan GD , Bhat BA , Wahl LM , Agashe D . Shifts in mutation spectra enhance access to beneficial mutations. bioRxiv . 2022:284158. 10.1073/pnas.2207355120 PMC10235995 37216547

[43] Ibrahim A , Fros J , Bertran A , Sechan F , Odon V , Torrance L , et al. A functional investigation of the suppression of CpG and UpA dinucleotide frequencies in plant RNA virus genomes. Sci Rep. 2019;9:18359. 31797900 10.1038/s41598-019-54853-0 PMC6892864

[44] Atkinson NJ , Witteveldt J , Evans DJ , Simmonds P . The influence of CpG and UpA dinucleotide frequencies on RNA virus replication and characterization of the innate cellular pathways underlying virus attenuation and enhanced replication. Nucleic Acids Res. 2014;42:4527–45. 24470146 10.1093/nar/gku075 PMC3985648

[45] Odon V , Fros JJ , Goonawardane N , Dietrich I , Ibrahim A , Alshaikhahmed K , et al. The role of ZAP and OAS3/RNAseL pathways in the attenuation of an RNA virus with elevated frequencies of CpG and UpA dinucleotides. Nucleic Acids Res. 2019;47:8061–83. 31276592 10.1093/nar/gkz581 PMC6735852

[46] van Dorp L , Richard D , Tan CCS , Shaw LP , Acman M , Balloux F . No evidence for increased transmissibility from recurrent mutations in SARS‐CoV‐2. Nat Commun. 2020;11:5986. 33239633 10.1038/s41467-020-19818-2 PMC7688939

[47] Shu Y , McCauley J . GISAID: global initiative on sharing all influenza data—from vision to reality. Euro Surveill. 2017;22:30494. 28382917 10.2807/1560-7917.ES.2017.22.13.30494 PMC5388101

[48] Robinson JT , Thorvaldsdóttir H , Winckler W , Guttman M , Lander ES , Getz G , et al. Integrative genomics viewer. Nat Biotechnol. 2011;29:24–6. 21221095 10.1038/nbt.1754 PMC3346182

[49] Cingolani P , Platts A , Wang LL , Coon M , Nguyen T , Wang L , et al. A program for annotating and predicting the effects of single nucleotide polymorphisms, SnpEff: SNPs in the genome of *Drosophila melanogaster* strain w1118; iso‐2; iso‐3. Fly. 2012;6:80–92. 22728672 10.4161/fly.19695 PMC3679285

[50] O'Toole Á , Scher E , Underwood A , Jackson B , Hill V , McCrone JT , et al. Assignment of epidemiological lineages in an emerging pandemic using the pangolin tool. Virus Evol. 2021;7:veab064. 34527285 10.1093/ve/veab064 PMC8344591

[51] Stamatakis A . RAxML version 8: a tool for phylogenetic analysis and post‐analysis of large phylogenies. Bioinformatics. 2014;30:1312–3. 24451623 10.1093/bioinformatics/btu033 PMC3998144

[52] Price MN , Dehal PS , Arkin AP . FastTree: computing large minimum evolution trees with profiles instead of a distance matrix. Mol Biol Evol. 2009;26:1641–50. 19377059 10.1093/molbev/msp077 PMC2693737

