# Supplementary material for: Massive‐scale genomic analysis reveals SARS‐CoV‐2 mutation characteristics and evolutionary trends
Source: mLife. 2022 Sep 26;1(3):311–22. doi: 10.1002/mlf2.12040 (PMC9538474; doi:10.1002/mlf2.12040)
Supplement: Supplementary file 1 — Supporting information. [file MLF2-1-311-s001.docx]

**Supplemental Information for:**

**[Massive-scale genomic analysis reveals](https://www.projecttopics.org/massive-scale-genomic-study-reveals-wheat-diversity-for-crop-improvement.html) SARS-CoV-2 mutations characteristics and evolutionary trends**

Names of all Authors as they appear on the manuscript

**Figure legends:**

**Figure S1** Comparison of mutation frequency between all sites and 4DTV sites.

**Figure S2** Comparison of mutation frequency of different 12 dinucleotide (CG, GC, AT, TA, TC, CT, AG, GA, CA, AC, TG, and GT)

**Figure S3** The mutation frequency distribution of non-CG, non-AM and non-TA sites at 4DTv.

**Figure S4** The frequency of SARS-CoV-2 codon usage (A). Proportion of A, T, C and G bases in the third position of codon (B).

**Figure S5** Comparison of mutation spectrum between humans and *Neovison vison.*

**Figure S6** The flowchat of evolutionary tree construction.

**Figure S7** The flowchat of DNMs detaction.


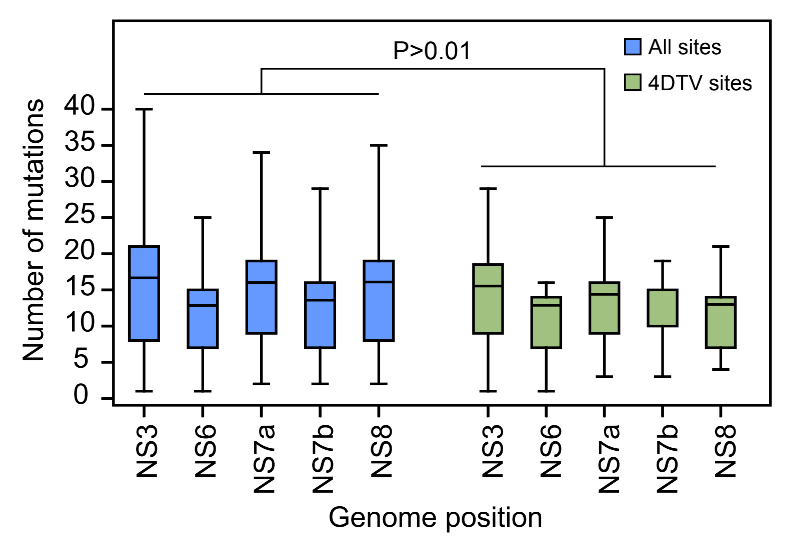


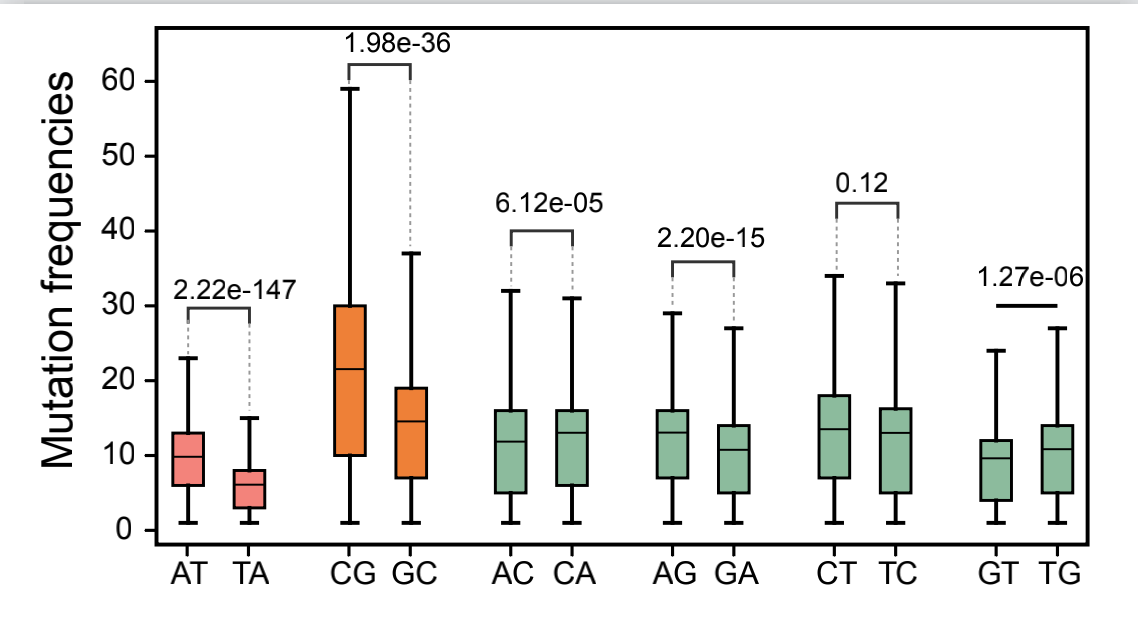


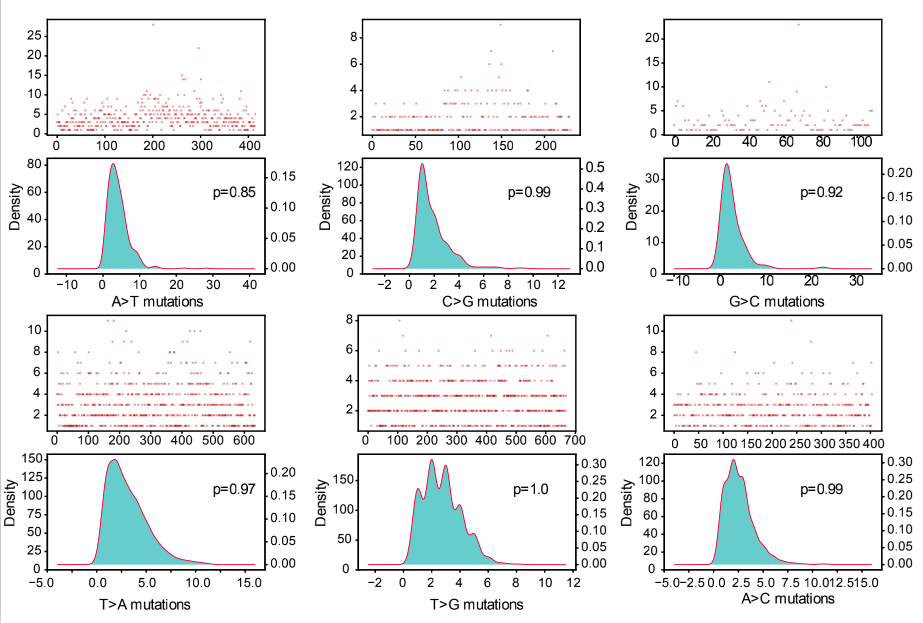


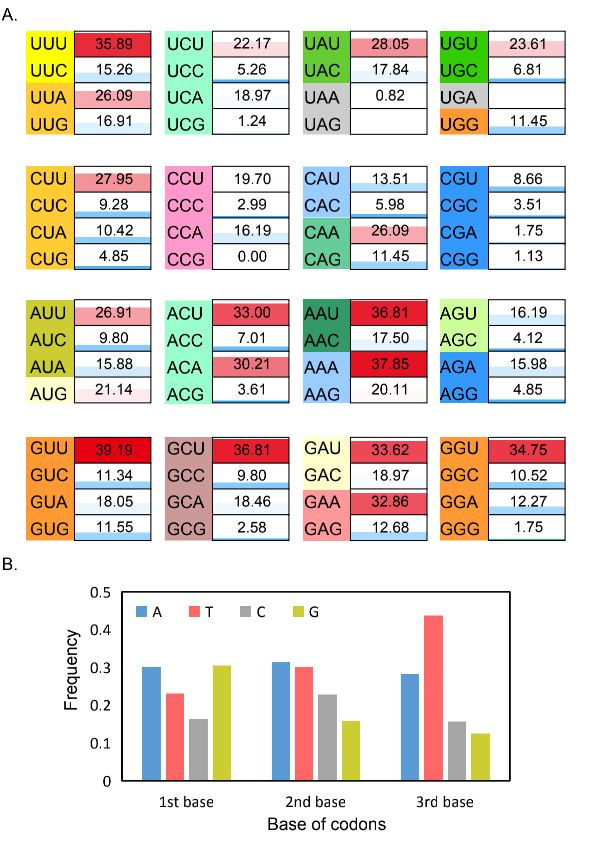


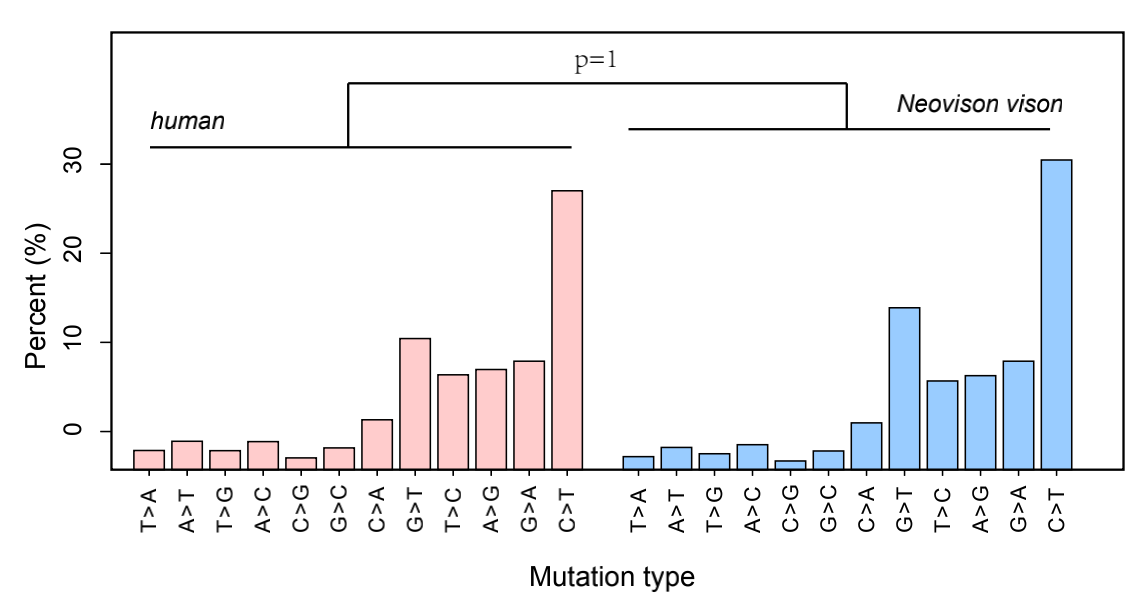


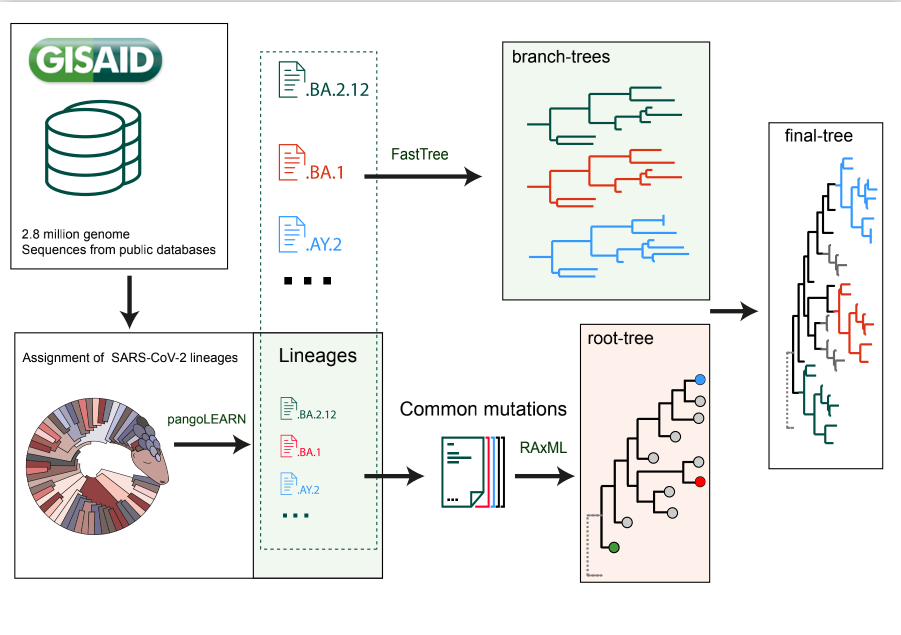


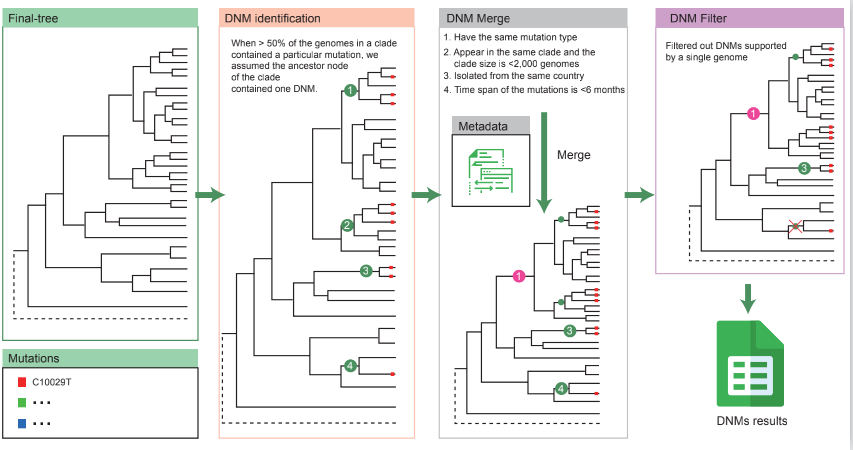


Table S1 Comparison of the mutation spectrum at 4DTv, 5′-[UTR](https://cn.bing.com/search?q=Untranslated+region&filters=sid:376d069b-9e2a-cbba-d833-321a47870605&form=ENTLNK) and 3′-[UTR](https://cn.bing.com/search?q=Untranslated+region&filters=sid:376d069b-9e2a-cbba-d833-321a47870605&form=ENTLNK) of non-CG, non-AM, and non-TA. (The numbers marked in red represent proportion of C and G mutations into T were highest at 4DTv sites)

|  | C->T | C->A | C->G | G->T | G->A | G->C |
| --- | --- | --- | --- | --- | --- | --- |
| 4DTv | **0.7902** | 0.1737 | 0.0361 | **0.6243** | 0.3087 | 0.0670 |
| 5'-UTR | 0.7132 | 0.1860 | 0.1009 | 0.5047 | 0.3863 | 0.1090 |
| 3'-UTR | 0.7530 | 0.1747 | 0.0723 | 0.5148 | 0.3040 | 0.1812 |

Table S2 The dinucleotide mutation frequency in 1 + 2, 2 + 3, 3 + 1 codon phase

|  | all sites | | 1st+2nd | | 2nd+3rd | | 3rd+1st | |
| --- | --- | --- | --- | --- | --- | --- | --- | --- |
|  | Mutation frequency | Ratio of frequency | Mutation frequency | Ratio of frequency | Mutation frequency | Ratio of frequency | Mutation frequency | Ratio of frequency |
| AT/TA | 9.83/6.10 | 1.61 | 10.2/8.5 | 1.20 | 9.26/8.73 | 1.06 | 10.29/6.84 | 1.51 |
| AG/GA | 13.06 /10.76 | 1.21 | 9.89/1182 | 0.84 | 12.85.9.58 | 1.34 | 12.55/13.83 | 0.91 |
| GT/TG | 9.63/10.84 | 0.89 | 11.34/7.84 | 1.45 | 8.75.12.32 | 0.71 | 13.3/10.54 | 1.26 |
| AC/CA | 11.86/13.04 | 0.91 | 12.24/10/73 | 1.14 | 13.39/12.25 | 1.09 | 10.69/15/50 | 0.69 |
| CT/TC | 13.53/13.03 | 1.03 | 14.16/11.93 | 1.19 | 12.18/15.47 | 0.79 | 15.9/13.88 | 1.15 |
| CG/GC | 21.52/14.56 | 1.48 | 13.56/13.61 | 1.00 | 21.41/14.91 | 1.44 | 22.91/17.07 | 1.34 |
